# Hybrid and Endovascular Management of Aortic Arch Pathology

**DOI:** 10.3390/jcm13206248

**Published:** 2024-10-19

**Authors:** Richard Shi, Mathew Wooster

**Affiliations:** Department of Vascular Surgery, Medical University of South Carolina, Charleston, SC 29425, USA; shir@musc.edu

**Keywords:** aortic arch pathologies, thoracic endovascular aortic repair, aortic arch aneurysm, aortic arch dissection, aortic stent graft

## Abstract

The advent of endovascular aortic surgery has led to the rise of novel techniques and devices in treating pathologies of the aorta. While endovascular surgery has been well established in the descending thoracic and abdominal aorta, the endovascular treatment of the aortic arch represents a new and exciting territory for aortic surgeons. This article will discuss the different aortic diseases amenable to endovascular treatment, currently available aortic arch stent grafts and their limitations, and the future of endovascular aortic arch therapies.

## 1. Introduction

Pathologies of the aortic arch represent a unique challenge to cardiac and vascular surgeons and are increasingly being identified amongst the aging population. The gold standard in treatment is open arch surgery, but despite advancements in surgical techniques and cardiopulmonary bypass, mortality and stroke rates continue to be high (3.4% in elective repair, 15.4% in non-elective cases) [1]. Furthermore, surgery is prohibited in a large proportion of patients; over a quarter of patients with type A dissections are not offered surgeries due to significant comorbidities [2].

Advanced endovascular therapies have expanded the boundaries of treating aortic arch pathologies in a minimally invasive approach over the last decade. However, compared to descending aortic aneurysms, there are unique challenges to the arch which have prevented endovascular interventions from becoming mainstream. The principles of optimal medical therapy will always remain paramount to good patient outcomes. While open surgery represents the historic gold standard in this arterial space, we will focus on the burgeoning role of hybrid and purely endovascular techniques.

## 2. Defining Aortic Arch Pathologies

The most common aortic arch conditions that can be treated through endovascular techniques include aneurysms, pseudoaneurysms, acute aortic syndromes, and iatrogenic trauma.

### 2.1. Aortic Arch Aneurysms

Aneurysms are defined as the dilatation of all three layers of the vessel wall, to a diameter 50% greater than the normal aortic diameter. The incidence of thoracic aortic aneurysms is around 10 cases per 100,000 person-years, with aortic arch aneurysms representing around 10% of all thoracic aneurysms [3]. The most devasting complication is rupture, with an annual rupture risk of 6.9% for aneurysms exceeding 6 cm [4].

The etiology and pathophysiology of aortic arch aneurysms are varied. Atherosclerosis is the most common etiology: underlying atheromas contain inflammatory cells that release mediators that accelerate the breakdown of matrix protein. The vaso vasorum is also impaired, leading to degenerative, ischemic changes in the aorta [5]. Thus, risk factors include hypertension, hyperlipidemia, and smoking. Other etiologies include connective tissue disorders (Marfan syndrome, Loeys–Dietz, Ehlers–Danlos) and infectious or non-infectious inflammatory conditions. Such conditions result in medial degeneration and the subsequent weakening of the aortic wall, leading to aortic aneurysm formation [6]. These etiologies represent 80% of all thoracic aortic aneurysms; chronic dissections with aneurysmal degeneration represent the other 20%.

### 2.2. Acute Aortic Syndromes

Acute aortic syndromes (AASs) represent a triad of conditions, including acute aortic dissection (AAD), intramural hematoma (IMH), and penetrating aortic ulcers (PAUs). Typical symptoms include sudden and severe ripping/tearing chest or back pain. AADs account for 80% of AASs and involve an intimal tear in the aorta which creates a true and false lumen. A total of 65% of intimal tears are found in the ascending aorta, 10% in the aortic arch or abdominal aorta, and 25% in the descending thoracic aorta [7]. These flaps can result in the static or dynamic obstruction of any aortic branch, resulting in malperfusion and necessitating urgent intervention. In the arch, these could lead to stroke, myocardial infarction, and spinal cord ischemia. The incidence of AAD is around 3.0 per 100,000 person-years, and the major risk factors include bicuspid aortic valve, connective tissue disorders, cocaine abuse, and hypertension [8].

PAUs and IMHs are variants of aortic dissection and are present in one-eighth of all patients with AAD [9]. PAUs are focal ulcerations of the intima and media that can degenerate into pseudoaneurysms and saccular aneurysms; they are present in 2–8% of those with AAS [10]. IMHs are caused by the rupture of the vaso vasorum and bleeding into the aortic media and are present in 5–20% of patients with AAS. IMHs frequently occur in conjunction with PAUs; their joint appearance results in a high risk of progression to dissection or rupture [11,12]. In general, unless symptomatic or with signs of impending rupture or progression, most PAUs and IMHs should resolve with medical management such as strict blood pressure control and close imaging follow-up [13].

### 2.3. Pseudoaneurysms

Pseudoaneurysms in the aortic arch are defined by the disruption of all three vessel walls of the aorta, leading to a focal dilatation that is only contained by periadventitial connective tissue. Pseudoaneurysms can occur due to atherosclerosis or PAUs, trauma, or post-surgical iatrogenic injury. Such patients will present with chest pain and signs of compression by the pseudoaneurysm, such as heart failure, valve failure, stroke, or tracheal compression. A previous open surgery of the thoracic aorta is the most common cause of pseudoaneurysms [14]. Pseudoaneurysms can transpire at previous anastomosis sites, aortotomy sites, and cannulation sites. Studies have shown infection (endocarditis or mediastinitis) to be a significant contributor of pseudoaneurysm formation at these sites [15].

Blunt trauma to the aortic arch can also cause pseudoaneurysms, due to the acceleration–deceleration forces from falls or car accidents. This shear force is the greatest at the aortic isthmus and will cause the ligamentum arteriosum to rupture. Such injuries are commonly treated through endovascular repair, although in the setting of trauma, other concurrent injuries impact the final treatment options [16].

## 3. Modern Techniques for the Hybrid Repair of the Aortic Arch

Open aortic arch surgery is the standard for the repair of aortic arch aneurysms and dissections but faces high rates of mortality and neurologic complications. The complexity and morbidity of these procedures led to the advent of hybrid arch repairs. The first open arch autologous debranching with stent graft placement for a ruptured arch aneurysm was described in 2003 by Czerny and colleagues [17]. There have been a variety of techniques established since.

**Figure 1:**
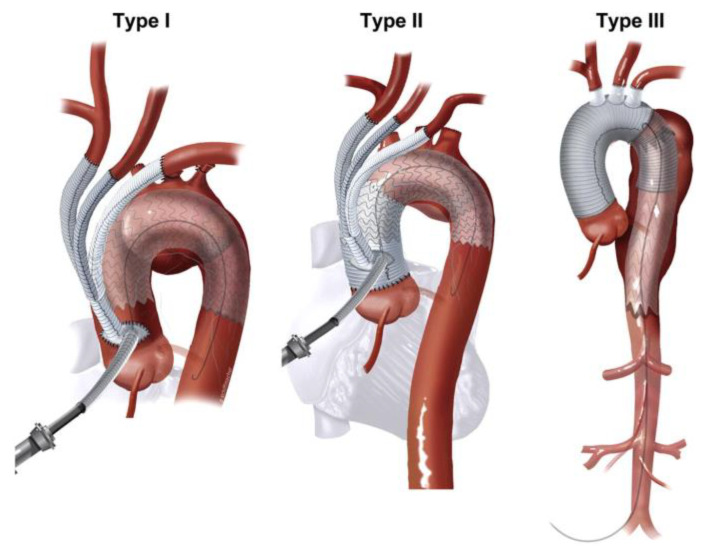
Hybrid arch repair classification system developed by Bavaria et al. Type I: Debranching via multi-branched Dacron graft sewn to native ascending aorta with subsequent TEVAR deployment. Type II: Dacron replacement of ascending aorta with surgical debranching onto graft with TEVAR deployment. Type III: Total arch replacement with descending elephant trunk and delayed retrograde TEVAR [18].

Bavaria et al. proposed a hybrid arch repair classification system consisting of three types [18]. The first type is a surgical debranching of the aortic arch vessels via a multi-branched Dacron graft that is sewn to healthy native ascending aorta and is performed in disease limited to the aortic arch. This is conducted via a full or partial sternotomy, although when the LSA is not accessible, a carotid–subclavian bypass/transposition is performed. After debranching, a TEVAR is delivered retrograde/anterograde, in a staged or combined approach. This technique requires at least a 2 cm proximal landing zone. The second type is where a portion of unhealthy ascending aorta and/or aortic arch is replaced with a Dacron graft with distal anastomosis at zone 1 or 2 and debranching onto the graft. This allows for 4–6 cm of a proximal seal zone for the TEVAR. The third type is akin to the conventional elephant trunk, where the aortic arch is replaced with a Dacron graft sewn to the distal native aortic arch, with a downstream portion that floats free within the descending thoracic aorta. The arch vessels are reimplanted onto the Dacron graft. This method allows for a longer seal zone, and a TEVAR is landed within the free-floating graft (Figure 1). However, these procedures still carry risk, as a systematic review of 1886 hybrid arch procedures demonstrated a pooled mortality rate of 10.8%, peri-operative stroke rate of 6.9%, and spinal cord ischemia rate of 6.8% [19].

**Figure 2:**
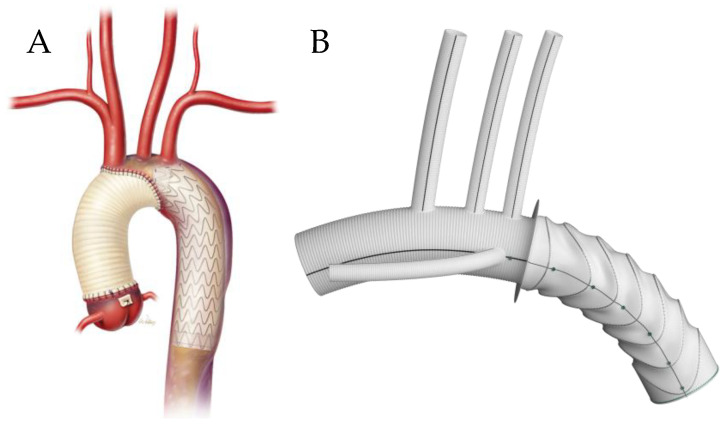
(**A**) Ascending aorta and proximal arch replacement with antegrade stent graft delivery [22], (**B**) four-branch Thoraflex Hybrid device, courtesy of Terumo Medical [23].

An advancement of the conventional elephant trunk is the frozen elephant trunk (FET), a single-stage, hybrid technique that is now the gold standard for acute type A aortic dissections. It was introduced in 2003 by Karck and colleagues and is frequently described in conjunction with a hemiarch repair [20]. In this procedure, the ascending aorta and lesser curvature of the aorta are resected, leaving the arch vessels intact. This allows for the antegrade delivery of a TEVAR in the true lumen to cover any distal entry tears. The distal Dacron graft is anastomosed to the native aorta and stent graft along the lesser curvature. The self-expandable stent graft is frozen in place within the aortic lumen, allowing for false lumen thrombosis and aortic remodeling (Figure 2A). The FET can also be used with total arch replacement, when intimal tears originate at the aortic arch or involves the arch branches. In this version, all arch vessels are resected and anastomosed onto a proximal Dacron graft. In a similar fashion, the TEVAR stent graft is advanced anterograde in an open surgical field and incorporated into the distal anastomosis suture line.

The Thoraflex Hybrid graft (Terumo Aortic, Glasgow, Scotland, UK) is the only FDA-approved FET device available in the US, approved in 2022 (Figure 2B). The device is composed of a 4-branch aortic arch prosthesis connected to a Dacron stent graft. It allows for a single-stage repair of the ascending aorta, aortic arch, and proximal descending aorta. Three branches are used for arch vessel reconstruction, while the fourth is used for early reperfusion. This device is delivered under circulatory arrest. The aortic arch is resected, and the Thoraflex Hybrid is advanced via a guidewire into the descending thoracic aorta. The self-expanding stent graft portion of the device is deployed, and the cuff is anastomosed to the aorta, with individual branches anastomosed to their respective arch vessels. The proximal end is anastomosed to the sinotubular junction of the ascending aorta.

The Thoraflex device had promising results in a 1-year US-based multicenter trial. Amongst 12 US sites, 65 patients underwent open surgical repair with this device to treat aneurysms and/or dissections of the ascending aorta, aortic arch, and descending aorta. The study described an 81% one-year freedom from major adverse events and seven (11%) deaths. Permanent stroke and paraplegia/paraparesis occurred in 5% and 5%, respectively. A total of 26 (41%) patients had to undergo additional extension procedures of the distal aorta [21].

## 4. Commercially Available Devices in the United States

### Gore TAG Thoracic Branch Endoprosthesis

**Figure 3:**
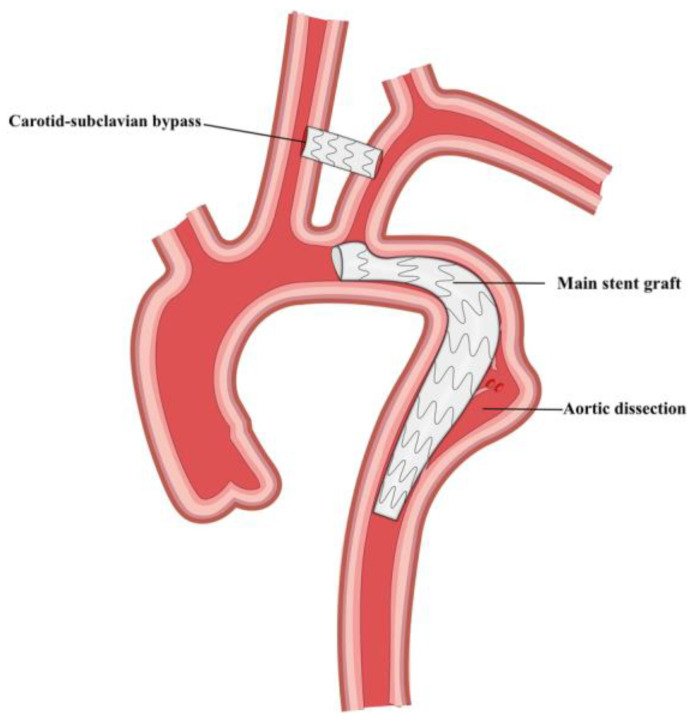
An example of a left carotid–subclavian bypass in conjunction with a TEVAR covering the left subclavian artery [27].

Thoracic endovascular aortic repair (TEVAR) has become the predominant surgical option in treating aneurysms, pseudoaneurysms, and dissections of the descending thoracic aorta. TEVARs require at least a 2 cm proximal seal zone to prevent endoleaks. However, up to 40% of descending thoracic aortic aneurysms will extend to the distal aortic arch or more proximal, requiring the covering of the LSA [24]. In an elective setting, LSA revascularization is indicated to reduce the risk of stroke, left upper extremity ischemia, and spinal cord ischemia [25]. Traditionally, this is conducted with a left common carotid artery (LCA) to LSA bypass (Figure 3). However, the risks include graft thrombosis, infection, nerve injury, thoracic duct injury, and bleeding [26]. Alternative endovascular options for zone 2 TEVARs involve off-label techniques (in situ laser fenestration, parallel grafts) or, more recently, branched stent grafts.

**Figure 4:**
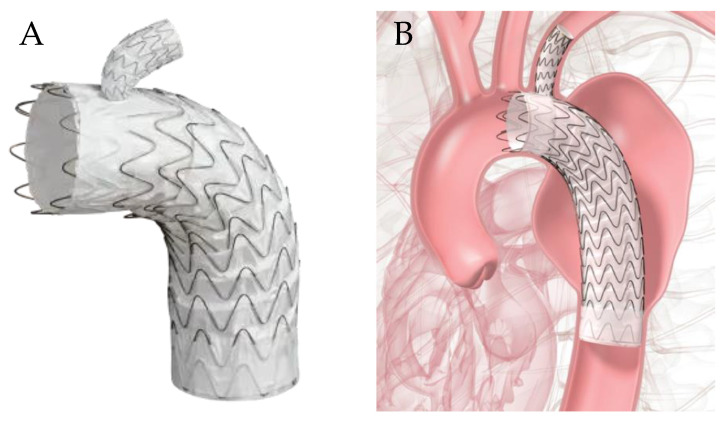
(**A**) Gore TAG Thoracic Branched Endograft, (**B**) TBE within aneurysm. Courtesy of Gore & Associates [34].

The Gore TAG Thoracic Branch Endoprosthesis (TBE; W.L. Gore & Associates, Inc.: Newark, DE, USA) is an aortic endograft with a single side branch for the LSA, designed to treat aneurysms/dissections requiring a zone 2 TEVAR (Figure 4). It is the only commercially available device for use in the US and was approved by the Food and Drug Administration (FDA) in 2022. The TBE is composed of up to three components: the main aortic component (AC), a side branch (SB) component, and an optional aortic extender. In this procedure, guidewire access into the LSA and ascending aorta is obtained. The aortic wire is inserted into the main AC delivery device, while the branch wire is inserted into a removable guidewire tube that traverses through the SB portal and out the distal edge of the AC. The AC delivery device is then advanced over both wires and subsequently deployed with the branch portal facing the LSA. The SB stent is then delivered into the LSA.

In the indications for use, the landing zones cannot be aneurysmal, dissected, heavily calcified, or thrombosed. There must be 15–36 mm of length from the distal edge of the LSA to the LCA ostium. The inner aortic diameter must range between 16 and 42 mm, and the LSA inner diameter must range from 6 to 18 mm. Furthermore, the outer curve length to the AC must be >2 cm proximal to the celiac artery [28].

The TBE underwent an investigational device exemption clinical study from 2016 to 2019 to evaluate its safety and effectiveness for treating zone 2 patients. The trial was a non-randomized prospective study representing 34 clinical sites and 246 patients. The aneurysm arm of the study had 84 patients. Technical success occurred in 91.7% of patients; three patients had inaccurate deployment, and four had unanticipated additional intra-operative procedures. There four 4 patient deaths in the cohort, although all were unrelated to the device or procedure. Three patients (3.6%) developed periprocedural stroke, two patients (2.4%) developed spinal cord injury, seven patients (8.3%) had a new dissection (one retrograde type A), and four patients (4.8%) experienced iliac artery rupture during delivery. Over one year, endoleaks were found in eight patients (9.8%): three type Is and five type IIIs. Only one type III endoleak required re-intervention, which was the only re-intervention for the entire study. In the dissection arm, 132 patients were enrolled. Technical success was higher at 97.7%, although there was one case of aortic rupture and three cases of lesion-related mortality. There were six patients that required re-intervention. Procedure and treatment success in the dissection cohort group was 83.3% and 89.8% in one year [28,29].

There have also been several non-clinical trial, institutional studies. Squiers et al. performed a retrospective review on patients that received a TBE or surgical revascularization. The TBE was associated with less operative time and a shorter length of stay compared to surgical revascularization, although there was no difference in operative mortality and survival at 3 years out [30]. Other studies report high technical success and patency with relatively limited minor complications [31,32].

The TBE remains under investigation for zone 0 and 1 aneurysms. Dake et al. presented preliminary data on zone 0 and 1 TBEs with nine clinical trial patients, revealing 100% successful device deployment and branch patency, with no endoleaks and two deaths at the one-year follow-up (one from hypoxic ischemic encephalopathy, the other from acute respiratory failure). Two patients developed periprocedural strokes and an additional stroke at the one-year follow-up. Of note, the patient who suffered a stroke was the same patient who passed from hypoxic ischemic encephalopathy [33].

## 5. Commercially Available Devices Outside of the United States (OUS)

### 5.1. Endospan Nexus

**Figure 5:**
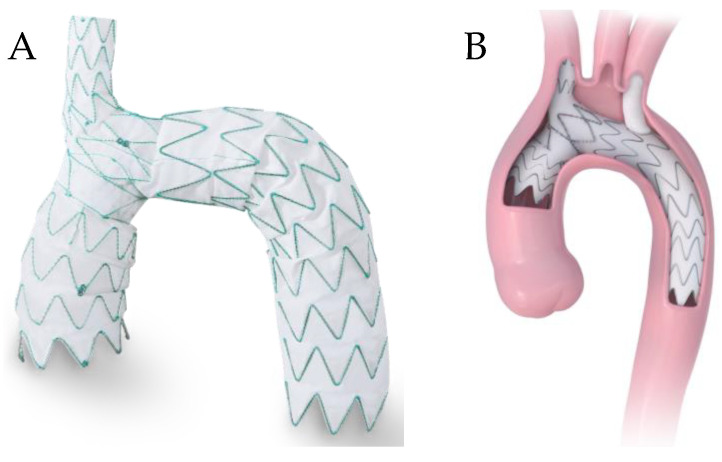
(**A**) Endospan Nexus device with arch stent and ascending stent graft combined, (**B**) Nexus duo device within an aneurysm. Courtesy of Endospan [40].

The NEXUS Aortic Arch Stent Graft System (Nexus; Endospan LTd., Herzliya, Israel) is a CE mark-approved off-the-shelf device for the endovascular repair of aneurysms and dissections of the ascending aorta and arch. The Nexus is a single-branch device consisting of two components: an ascending stent graft and an arch stent graft, coupled with a distal extension as necessary (Figure 5A). The ascending stent features unique distal bare metal struts which lock to an internal ring in the proximal arch stent graft. The stent graft features an integrated brachiocephalic artery (BCA) branch, requiring a right common carotid to left common carotid to left subclavian artery bypass. Furthermore, the delivery device is curved to meet the curvature of the arch and is delivered through a 20 Fr sheath. Anatomically, the proximal and distal aorta and BCA landing zones must be free of disease, the iliac/femoral arteries must be greater than 7 mm, and the right axillary artery must be greater than 3 mm [35].

The procedure involves femoral and axillary artery access for through-and-through wire access, with venous access for right ventricle pacemaker lead placement. The Nexus is delivered via the through-and-through wire until the proximal struts of the BCA branch are just proximal to the RCA origin and deployed. Subsequent wire access into the ascending aorta is obtained, and the ascending stent graft is delivered under rapid pacing. Lastly, a kissing balloon angioplasty of the brachiocephalic artery branch and the ascending stent graft is performed [36].

Two major studies investigated the one- and three-year safety and performance of the Nexus Endospan. These studies featured 28 patients: 17 patients (60.7%) had isolated arch aneurysms, 6 patients (21.4%) had chronic aortic dissection, and the rest had PAUs or combined pathologies. All patients had a proximal landing of the stent graft into zone 0. All procedures were completed successfully, and 10 patients had a planned parallel stent graft to the LSA. Two patients had procedure-related mortality within 30 days and another at one year, totaling three deaths (10.7%). As a secondary endpoint, freedom from major adverse events (all-cause mortality, myocardial infarction, new renal failure requiring dialysis, disabling stroke, and new aortic valve insufficiency) at 30 days was 89.2%. While no disabling stroke was reported, two patients (7.1%) had a stroke/transient ischemic attack at one year. Two (7.1%) patients demonstrated type III endoleaks at 30 days, with one resolving at one year [36]. At the three-year follow-up, there was a 71% overall survival of 25 patients. The percentage of unplanned re-intervention at 1 and 3 years was 11% and 29%, respectively, related to LSA stent relining and TEVAR placement for distal disease progression. There were no major adverse events reported and no strokes detected. There were four new endoleaks between the one- and three-year follow-up period. Only one patient had an aneurysm expansion [37].

There are ongoing US clinical trials for the NEXUS device. The TRIOMPHE trial is a non-randomized, multicenter investigational device exemption (IDE) study involving 110 patients at 31 clinical sites with chronic dissections, aneurysms, and PAUs/IMHs. Out of the 110 patients, 60 have chronic dissection, 30 have aneurysms, and 20 have PAUs/IMHs. The trial is designed for a 5-year follow-up. The primary outcome measures are device technical failure (failed deployment, device occlusion, failed exclusion of entry tear, additional procedures) and clinical failure (early mortality or major adverse events) at 30 days. The study began on 20 October 2020 with an estimated completion date of 2029 [38].

The 30-day results of the first 22 patients enrolled in the TRIOMPHE study were recently presented at the Society of Thoracic Surgeons 60th Annual Meeting. There were two (9.1%) early mortalities, one in the chronic dissection group and one in the aneurysm group. There was no disabling stroke/paraplegia or renal failure. The mean intensive care unit stay was 3.4 days, and the mean hospital stay was 10.6 days. As of 2024, the trial is nearly finished enrolling patients [39].

In order to reduce the perceived risks associated with the entire cerebral circulation originating from a single branch, Endospan released the custom-made Nexus Duo arch stent graft, which features a second branch channel for the stenting of the LSA (Figure 5B). This configuration reduces the number of vessels to be bypassed, requiring only a carotid-to-carotid or subclavian-to-carotid bypass. However, unlike the Nexus, the Duo requires through-and-through wire access from the femoral arteries to the bilateral axillary arteries. This device is currently being investigated in Europe. 

### 5.2. Terumo Relay Arch Branch

**Figure 6:**
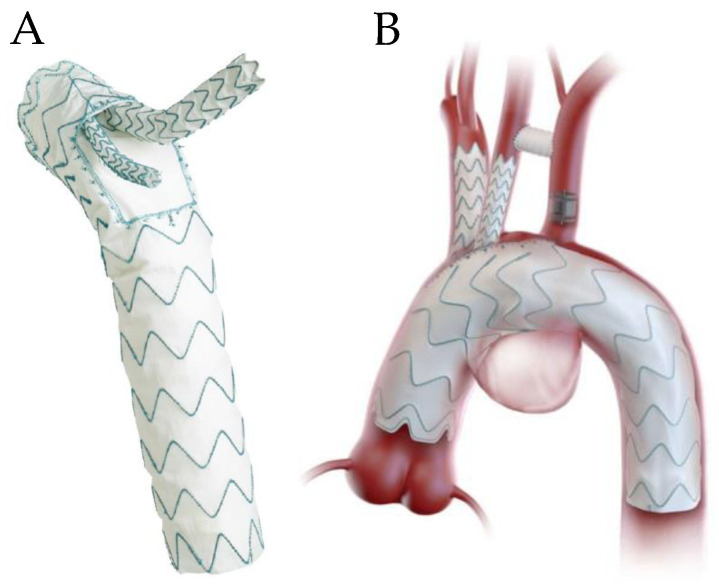
(**A**) Terumo Relay Branch device with two branches, (**B**) device with zone 0 placement with innominate artery and left common carotid branch, with carotid subclavian bypass. Courtesy of Terumo Aortic [40].

The Terumo Relay Branch (Terumo Aortic, Sunrise, FL, USA) is designed for zone 0 placement. There are multiple configurations available, but the off-the-shelf version consists of a main body stent graft that is deployed into zone 0 and a large cannulation window with two tunnels that allow for stenting into the BCA and LCA (Figure 6). The delivery system is pre-curved to align with the aortic arch curvature. The device is available in Europe and Asia, with over 300 patients treated over the last 3 years. In addition to the off-the-shelf design, custom designs with single, double, and triple branches can be ordered.

The off-the-shelf device, like other endovascular arch options, requires extra-anatomical bypass for the revascularization of the LSA. The device is advanced and deployed from femoral access under rapid pacing and confirmed to be appropriately positioned via angiogram. The bilateral common carotid arteries are accessed under open exposure, and BCA and LCA stents are delivered retrograde after the cannulation of both tunnels.

Several single/multi-institution studies have reported on this device. Kudo et al. performed a retrospective single-center study in 2020 for 28 patients which found a peri-operative stroke rate of 14.3% but no endoleaks or aortic events within 30 days. In the long-term follow-up, the survival rate at 1, 3, and 5 years was 92.7%, 85.6%, and 80.8%, respectively. Only two patients had aneurysmal enlargement, with the rest having no change in size or shrinkage [41]. In 2023, Jubouri et al. presented a multi-center international study on the double- and single-branch Relay in 125 patients. In the single-branch Relay group, there were no mortalities, strokes, or interventions within a 24-month follow-up period. These outcome rates were higher in the double-branch group although they were not statistically significantly different than those of the single-branch group [42].

The RelayBranch device was given the FDA Breakthrough Device Designation to fast-track through the regulatory review process, as part of a feasibility study for the dual-branch design that is currently enrolling patients in the US. Clinical trials are still ongoing.

### 5.3. Cook Medical Zenith Arch Branch Device

**Figure 7:**
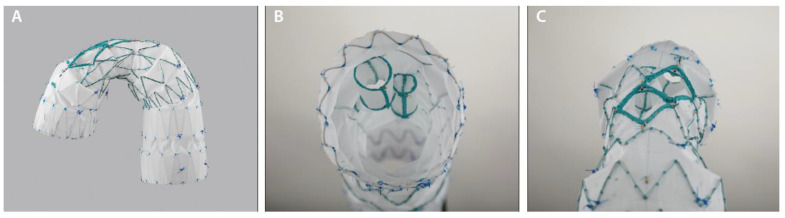
(**A**) Zenith arch branched device. (**B**) Internal side branches. (**C**) External view of side branches along outer curve of graft. Courtesy of Cook Medical [47].

The Zenith arch branch device (Cook Medical, Brisbane, Australia) is a custom-made device used to treat aortic aneurysms and dissections requiring a zone 0 seal zone. Any combination from 1–3 branches or fenestrations can be created, but the off-the-shelf device design contains two internal side branches for the BCA and LCA with an enlarged external opening at each distal end (Figure 7). The BCA branch stent is manufactured by Cook, while the LCA branch stent is a separate stent. The graft is narrowed in the middle to facilitate the branch stents. The anatomic criteria require a seal zone of >40 mm length and <38 m diameter, innominate artery >20 mm seal zone length and <20 mm diameter, and iliac access to accommodate the 22/24 Fr sheath [43].

LSA revascularization is performed prior to graft deployment unless a three-branch device is selected. The device requires three arterial percutaneous or open access sites: one femoral, one right axillary or carotid, and one left axillary or carotid [44]. The main body of the graft is delivered and deployed through femoral artery access under rapid pacing. The side branches are cannulated from their respective access points, and bridging stents are deployed as well.

A multi-center study in France, Japan, and Germany was performed in 2014 for 27 patients with aortic arch aneurysms. They reported 100% success and 0 thirty-day post-operative mortality, although there were three total strokes (11.1%) [45]. Over a one-year follow-up period, one patient passed from a remote thoraco-abdominal aneurysm rupture, and there were three type 2 endoleaks (11.1%). Another larger, recent global feasibility study was performed in 2020, which looked at three vessel Zenith arch branch devices for arch aneurysms in 39 patients. The technical success rate was 100%, and the combined mortality and stroke rate was 8%. A total of 12% required re-intervention: half were access complications and the other half endoleaks. At one year, patient survival was 90% [46].

The Zenith arch branch device remains an investigational device in both the US and OUS and is not yet FDA- or CE mark-approved at this time.

### 5.4. Gore Ascending Stent Graft

Stenting the ascending aorta is a novel concept and unique challenge given the presence of the coronary arteries and aortic valve and the relatively short length of the aorta in which to land. The earliest off-label devices include the 10 cm Gore TAG Conformable Thoracic Stent Graft and proximal AAA aortic extender cuff grafts. Dissections with small entry tears or pseudoaneurysms have been the main pathologies treated [48].

**Figure 8:**
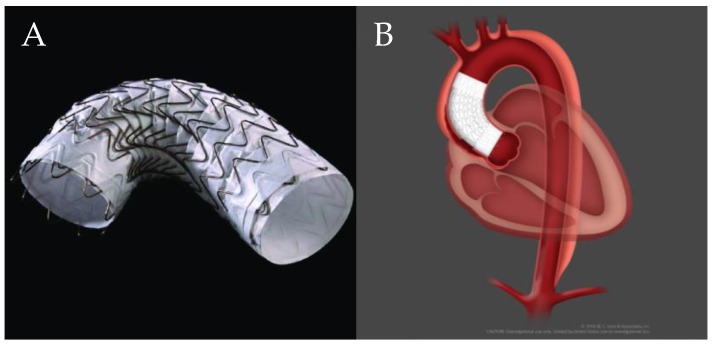
(**A**) Gore ascending stent graft, (**B**) within aneurysm. Courtesy of Gore & Associates [51].

The Gore ascending stent graft (W.L. Gore & Associates, Inc.: Newark, DE, USA) is a single stent designed for placement into the ascending aorta (Figure 8). It was studied under the ARISE trial which investigated its feasibility in treating type A dissections by sealing entry tears in the ascending aorta. It features a unique ability to partially deploy and shorten its inner curve orthogonal to the sinotubular junction to match the curvature of the arch. This feature was designed to prevent bird-breaking as seen in off-label TEVAR devices and help maintain adequate aortic wall apposition. The device is sized 6–33% over the diameter of the aorta at the sinotubular segment (proximal landing zone) and the aorta just proximal to the BCA (distal landing zone) and comes in lengths 24 mm to 42 mm.

The ARISE trial featured 19 patients from seven different clinical sites in the US. The inclusion criteria were DeBakey type I/II aortic dissection and entry tear in the ascending aorta at least 2 cm distal to the coronary artery. Their primary endpoint was all-cause mortality at 30 days which occurred in 15.8% of patients. Major adverse cardiovascular and cerebrovascular events occurred in 15.8% of patients, with disabling stroke in one patient (5.3%) and myocardial infarction in another patient (5.3%). There were two type Ia endoleaks, one type Ic endoleak, and one type III endoleak. These early feasibility results demonstrate the promise of endovascular solutions in treating ascending aortic dissections in a subset of patients with prohibitive surgical risk [49].

The ARISE II trial is currently under way in 20 US locations and is investigating the endovascular treatment of both chronic and residual type A dissection in the ascending aorta. The treatment of the first trial patient occurred in December 2023. The study is a non-randomized trial involving three parallel treatment arms: (1) ascending stent graft only, (2) ascending stent graft with TBE, and (3) open surgical repair. Their primary endpoint is technical success and safety (absence of aortic rupture, disabling stroke, new-onset renal failure requiring dialysis) [50].

## 6. Off-Label Treatments

Off-label treatments refer to the use of techniques and graft modifications performed on existing, off-the-shelf endografts to treat complex thoracic and abdominal aortic aneurysms. These treatment options are readily available, as opposed to custom-made grafts which require a 6- to 8-week development/delivery period.

### 6.1. Parallel Grafts

**Figure 9:**
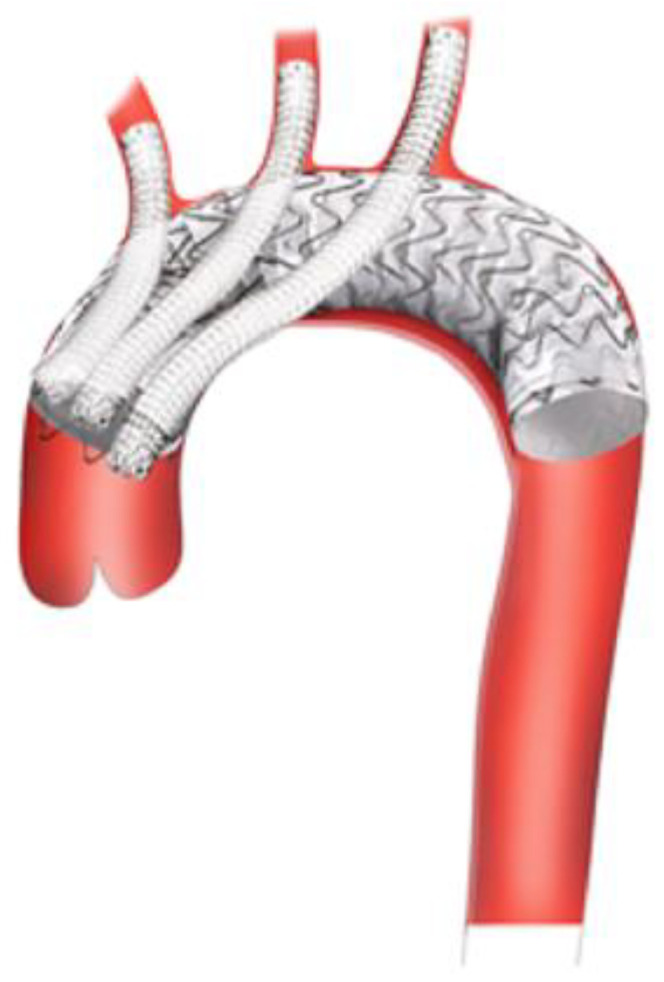
Ascending arch TEVAR with parallel grafts in all three arch vessels [52].

In parallel stent-grafting, branch vessel stent grafts are aligned parallel to the main aortic endograft (Figure 9). There are a variety of configurations (e.g., chimney, snorkel, periscope) depending on their position relative to the aortic endograft. In this technique, the aortic endograft is delivered through common femoral artery access, and branch stent grafts are delivered through the brachial or carotid arteries via percutaneous or open access techniques based on the number of branches required. The endograft is oversized around 10–20%, with the chimney graft at 0–5% oversizing. Furthermore, the parallel branch grafts should extend 1–2 cm beyond the aortic endograft [52].

Unlike the fenestration technique, parallel stent-grafting does not require physician modifications to the aortic endograft and is entirely off-the-shelf. One major disadvantage is the rate of endoleaks. The positioning of the branch grafts adjacent to the aortic graft enables blood flow between the grafts, causing type I endoleaks, termed “gutter” leaks. The placement of multiple parallel grafts results in higher rates of endoleaks. Shu et al. in a retrospective analysis on aortic arch endovascular treatments found an 11.1% type Ia endoleak rate in patients with the chimney technique, compared to 1% in patients with fenestrations [53]. Luo et al. performed a retrospective study on 32 patients with aortic arch pseudoaneurysms repaired with the chimney technique. There was one death within 30 days (3%), but over 3.5 years, the survival estimate was 84.4%. The patency of the branch vessels was 97.7%, and the endoleak rate was 9%, all of which were Ia endoleaks. None of the endoleaks required intervention [54]. Parallel grafting is a viable technique in treating aortic arch aneurysms, but careful intra-operative planning must be performed to limit endoleaks.

### 6.2. In Situ Laser Fenestration

**Figure 10:**
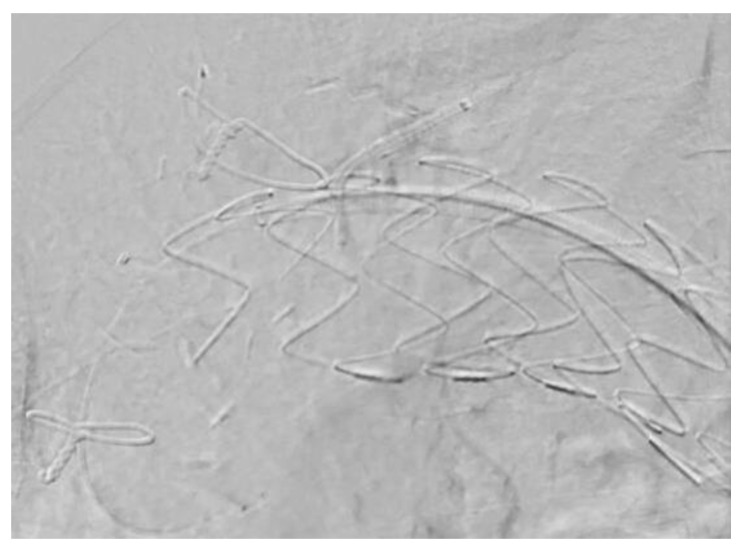
A laser catheter through the ostium of the left subclavian artery, inserting it in a perpendicular fashion through a TEVAR graft.

Laser fenestration has long been used in complex endovascular aortic aneurysm repair to preserve blood flow to branch vessels. This technique utilizes 308 nm UV wavelength light energy to create a 2–3 mm hole in the graft fabric, which can later be stented (Figure 10). It was first described in 2009 by Murphy et al., in which they performed a retrograde laser fenestration of a TEVAR via arm access to fenestrate the LSA during an acute aortic transection [55]. Laser fenestration is traditionally used in urgent situations such as aortic transection or ruptured aneurysms given its ability to rapidly exclude the aneurysm/pseudoaneurysm. The technique itself requires a perpendicular positioning of the laser tip to the aortic graft and is commonly conducted in the retrograde approach. The laser tip is slowly advanced with gentle pressure into the lumen of the aortic graft, and once through, a guidewire is advanced. This fenestration is balloon-dilated and subsequently stented.

There are some limitations to this technique. First, laser fenestration is not possible in branch vessels with severe angulation (<30 degrees) to the aortic arch, as this prevents the proper alignment of the laser probe tip to the aortic graft. Furthermore, there is a risk of type III endoleaks between the branch stent and fenestration, which in physician modified endografts (PMEGs) are reinforced with a metal ring. Additionally, this technique results in the coverage of the branch vessel while the fenestration is being performed, which can result in prolonged cerebral or upper extremity ischemia.

Houerou et al. performed a systematic review on in situ laser fenestration for the endovascular management of zone 0 to 2 aortic arch pathologies and found six retrospective studies with 247 patients. All three arch vessels were included in the study, with the predominant one being the LSA. They found a technical success rate of 98%, with a 3.2% death rate and 4.5% stroke rate within a 30-day follow-up period. The endoleak rate was 8% with a type III endoleak rate of 3.5% [56].

### 6.3. Fenestrated Physician-Modified Endografts

Physician-modified endografts (PMEGs) were born from the need to have an immediate solution to aortic pathologies using available off-the-shelf endografts. This technique involves the modification of a TEVAR on a surgical back table. Femoral access is required, alongside brachial and carotid access depending on the number of fenestrations. First, the TEVAR is partially unsheathed but remains attached to the delivery device. Computer tomography angiography is utilized to mark the distances and clock faces of the branch vessel fenestrations, which are created with cautery and reinforced with radio-opaque markers sutured in place. Diameter-reducing ties made of prolene are placed to help position and maneuver the device during deployment, and the device is resheathed. The device is delivered through femoral access, partially deployed, and positioned so that the fenestration markers meet the origins of the branch vessels. Fenestrations are then cannulated, and the device is fully deployed. Lastly, the branch vessels are stented in an order that minimizes cerebral ischemia [57].

There are several limitations of this technique. The first is the time required to plan the procedure and create fenestrations on the device, which may not be available in a patient with a rupture. Furthermore, there are structural concerns about man-made fenestrations in PMEGs and the incidence of type III endoleaks. Lastly, traditionally, there have not been many published data on this modality compared to other treatments described in this section, although more papers have been published in the last 2 years.

Wen et al. performed a recent retrospective study on 173 patients with a mix of aortic dissections, aneurysms, and PAUs/IMHs. There were a total of 20% of patients with three fenestrations, 19% of patients with two fenestrations, and 61% with one fenestration. The technical success rate was 98%, with a 2% rate of cardiac failure, renal injury, and stroke. There was a 4% death rate over an 11-month follow-up period. The endoleak rate was 6%, with a 6% re-intervention rate [58]. Similarly, Canaud et al. published a report on 100 patients with double-fenestrated TEVARs placed in zone 0. In this paper, fenestrations were created for the BCA, LCA, and LSA, but only the LSA was stented. The technical success rate was 95%, with a 2% 30-day mortality rate. The stroke rate was 4%, and the endoleak rate was 4%, with a re-intervention rate of 8% [59]. Overall, the short- and mid-term outcomes of PMEGs in the aortic arch are promising, but the long-term results will need to be assessed, particularly those of endoleaks and re-interventions.

### 6.4. Off-Label Endograft Use for the Ascending Aorta

The ascending aorta has been considered the next frontier for aortic endovascular techniques, of which there are no currently approved devices for. This area provides unique challenges given the presence of concomitant structures such as the aortic root and valve and the short length of the aorta compared to modern aortic stent grafts. On average, the length of the ascending aorta is 70–80 mm; commercially available TEVAR devices are too long. Furthermore, the ascending aorta is subject to higher pressures compared to the descending aorta, causing aortic diameter variability [60].

Roselli et al. studied 22 patients with TEVARs in the ascending aorta for pseudoaneurysms (from a prior cardiac anastomosis) and acute type A aortic dissections. Device delivery was transfemoral in 10 patients, transapical in 7, and transaxillary in 5. There were three peri-operative deaths. The 30-day, 1-year, and 5-year survival was 86%, 80%, and 75%, respectively. There were two coronary artery occlusions from the stent graft, which were both transfemoral, indicating the difficulty in accurate deployment with a longer delivery system. Furthermore, six patients developed endoleaks. Most devices used in this paper were aortic extension cuffs designed for the descending aorta. The authors note their preference for the 10 cm Conformable Gore TAG graft, which has a unique nested stent designed to allow for adaptability to the curvature of the aorta. However, most of the ascending aortas were too short [61].

Muetterties et al. performed a systematic review of ascending aorta stent grafts. They found that the most common indications for TEVAR in the ascending aorta are type A dissections, followed by pseudoaneurysms. The most used devices were existing TEVAR grafts, followed by aortic abdominal cuffs, followed by custom-made grafts. The most common vascular access point was the femoral artery, followed by the transapical and then the carotid artery. There was a significant type I endoleak rate of 18.6%, with 9.3% requiring re-intervention. There was a 3.4% stroke rate and a 3.4% post-operative myocardial infarction rate [62]. Overall, these studies highlight the benefit of an endovascular solution to the ascending aorta but showcase the severe design restrictions of the current options.

## 7. Discussion: Unique Challenges in the Arch

The ascending aorta and aortic arch present unique anatomical and physiological challenges which modern devices are not designed for. In this section, we will detail each one in depth and the current literature in how innovators have attempted to address them.

### 7.1. Measurements

Diameter measurements in the proximal and distal seal zone are imperative in graft sizing. The ascending aorta and aortic arch are subject to significant hemodynamic forces due to their proximity to the heart. Furthermore, the aorta is highly compliant due to elastic fibers in the media, resulting in expansion and contraction during systole and diastole. The ascending aorta can store up to 50% of the left ventricular volume during systole and is likened to a capacitator, termed the “Windkessel effect”. Studies have found an 8% aortic diameter variation during the cardiac cycle [63]. This variation emphasizes the importance of oversizing to prevent endoleaks and device migration. As a result, several papers recommend oversizing the aortic endograft at around 15–20%.

Another challenge is the placement of a TEVAR graft in a prosthetic graft, which is common given the prevalence of patients who have had a prior open aortic surgery. Unlike the native aorta, prosthetic grafts are more rigid and do not have the same diameter variation during the cardiac cycle [64]. As a result, the oversizing criteria for the native aorta are different in those with existing prosthetic grafts. Prendes et al. performed an in vitro study where they investigated different oversize ranges of a Zenith TX2 endograft with diameters between 24 and 36 mm deployed in a 24 mm Dacron graft. They determined that oversizing around 16% resulted in an optimal pull-out force (the force to pull the endograft from the PTFE graft) and that over 30% oversizing would result in graft infolding. The authors recommended around a 20–30% oversizing of a TEVAR within a Dacron graft, though most frequent implanters tend to oversize closer to 10–15% given concerns for infolding and the reduced elasticity of the graft [65].

Beyond diameter, the length of the aorta is also important. A 2 cm seal zone is required proximally and distally, and it is imperative to not encroach upon any important branch vessels. Due to the curvature of the aorta, there is a length discrepancy between the greater and lesser curvature and to any centerline distance. Thus, the TEVAR is sized to the greater curvature to ensure adequate seal zones. Iwakoshi et al. reviewed TEVARs placed for aortic arch aneurysms and utilized software to create virtual stent graft images based on the greater curvature, lesser curvature, and center lumen line based on the pre-operative scan. These images were then superimposed onto post-operative CT images. The greater curvature was the most accurate predictor of stent graft position [66].

Custom fenestrations must be carefully created based on pre-operative imaging, in which clock positions are determined based on every branch vessel. This is accomplished via centerline imaging when performing EVAR, but this is increasingly challenging in arch aneurysm repair as the current software is not as adequate. This is one reason that non-aligned branches are more frequently seen in current commercially available/trial devices.

### 7.2. Bridging Stents

Bridging stents are placed from the fenestrations/channels of the aortic graft to their respective arch vessels to maintain perfusion. To date, only the TBE endograft is designed with its own bridging stent. Alternatives include custom-made limbs, iliac limb devices from existing EVARs, or individual self-expanding, covered stents such as the Bard Fluency or Viabahn stent graft. The ideal bridging stent would have the longer length associated with self-expanding stents but the higher radial force and control of balloon-expandable stents. The stent itself must be able to take the large impulse forces and movement of the arch.

Furthermore, the stent itself should have large distal diameters and be oversized relative to the branch vessel but with a smaller diameter in the aortic graft to minimize the size of the fenestrations/branches created on the main body device. This minimizes the risks of fabric compromise, encroachment on stent architecture, and the volume of aorta filled by the branch stents.

Lastly, visualizing the distal landing zone from the proximal landing zone requires different obliquities of the C-arm. While the left common carotid artery is quite forgiving, the innominate artery is at very acute angles with short lengths, similar to the takeoff of the left vertebral artery. This limitation makes it difficult to achieve the maximum seal length of these branches without altering from the steep left anterior oblique preferred for identifying the aortic arch outer curve.

### 7.3. Nose Cone

Delivery systems for the aortic arch and ascending aorta are designed with a long nose cone to be able to track over the aortic arch. However, a long nose cone will cross into the aortic valve and left ventricle, which can result in valvular or ventricular trauma. This is especially a problem with mechanical valves, as the nose cone will interfere and cause aortic regurgitation [60]. A short nosecone allows for closer encroachment to the aortic valve but impairs trackability through the arch. In aortic valve devices, this has been overcome through coaxial and steerable sheaths, but these technologies require larger sheath sizes than would otherwise be required for the valve itself. This technology would be difficult to apply to TEVARs since the fabric of thoracic grafts inherently requires a larger sheath size than aortic valves and thus is limited by the diameter of iliofemoral access vessels.

### 7.4. Deployment

There are several considerations in the deployment of TEVAR in the ascending aorta and aortic arch. Given the high shear forces that the aortic endograft is subject to, decreasing cardiac output or inducing hypotension can facilitate precise graft deployment. The most common method is inserting a pacing catheter into the right ventricle to induce rapid ventricular pacing. However, pacing is not without risk, as an accelerated heart rate can result in hemodynamic changes that the patient may not tolerate. Other risks include cardiac perforation and tamponade and access site complications. A technique that does not require pacing is the use of a Valsalva technique to reduce cardiac output. Tsilimparis et al. developed the Munich Valsalva Implantation Technique, where a modified Valsalva maneuver is performed during mechanical ventilation to increase intrathoracic pressure, which compresses the vena cava and pulmonary veins, thereby reducing venous return [67]. While there are no hemodynamic changes typically associated with this technique, there have been reported increases in intracranial pressure and spinal fluid pressure, although the implications of this have been unclear. There are other techniques, such as pharmacologically induced hypotension or transient adenosine-induced cardiac arrest. However, the response is highly dose-dependent and can cause significant swings in blood pressure. Furthermore, these agents may have other harmful effects in the setting of concurrent renal, airway, and cardiac disease. Ultimately, rapid ventricular pacing is the most predictable and reliable technique amongst all those listed [68].

Another important consideration is the length of the delivery device. TEVARs are commonly deployed via femoral access and are designed for delivery in the descending thoracic aorta. Thus, the existing device lengths may not be sufficient for delivery into the aortic arch and ascending aorta, and different access points can be considered. Furthermore, a greater delivery length may impact deployment accuracy and device maneuverability [61]. Axillary access and transapical access have been shown as promising alternative methods given their shorter length of delivery [69]. However, there are limited data comparing the different modalities of access for TEVARs in the ascending aorta and aortic arch.

### 7.5. Stroke Rates

Stroke rates in the endovascular treatment of the ascending aorta and aortic arch are notably higher than those in the descending aorta. This is due to the crossing of the great vessels, the rotation of the device in the arch, and the potential of atheromatous and air embolic events during branch cannulation and endograft delivery. Furthermore, supra-aortic debranching for certain branched endografts contributes to stroke rates. A systematic review of different arch branch devices noted a stroke rate range from 5% to 25% with multi-branch devices and 3.6% with single-branch devices [70]. Stroke rates tend to increase when there is a greater number of branches and increase from zone 2 to zone 0 placement as well. One study showed a stroke rate of 11.1% in zone 0, 5.3% in zone 1, and 4.7% in zone 2 [71]. There are several methods to reduce stroke in endovascular repair, such as appropriate anticoagulation, consistent goal blood pressures, the adequate deairing of the stent graft, and embolic protection devices.

The rate of peri-operative stroke in open surgical aortic arch replacement in the literature ranges from 2% to 10% [1,72,73,74]. Both emboli from the thoracic aorta and cerebral hypoperfusion during cardiopulmonary bypass are causes of stroke during open repair. Overall, stroke rates in the endovascular repair of the arch are generally higher compared to those in open surgical repair, but this largely depends on the type of endovascular device used and zone landing.

### 7.6. Endoleak Rates

Endoleaks are a problem more unique to endovascular repair compared to traditional open surgical repair. The most common endoleaks are type I and type III. The use of the Gore TAG showed an endoleak rate of 9.8%, with three being type I and five being type III [29]. The use of the Endospan Nexus device resulted in two patients with a type III endoleak, contributing to a 7.1% endoleak rate [37]. When using the Terumo Relay branched endograft, an endoleak rate of 6.7% was reported [43]. When using the Zenith arch branch device, there was a 15% endoleak rate reported [46]. A literature review reported type IA endoleaks rates in off-label techniques and found a 20.1% endoleak rate in the chimney technique, a 5% endoleak rate in fenestrated stent grafts, a 2.3% endoleak rate in in situ fenestrations, and a 4.8% in branched endografts [75]. Thus, regardless of the technology or technique used, it is imperative to optimize endoleak rates for any endovascular aortic solution.

## 8. The Future: Endo-Bentall

While the ascending aorta is seen as the next frontier in endovascular aortic surgery, the endovascular repair of the aortic root may be seen as the last. Aortic root aneurysms and dissections have been traditionally treated with the Bentall procedure, where the aortic valve, root, and ascending aorta are replaced with a surgical graft, with the reimplantation of the coronary arteries into the graft. However, in acute type A aortic dissection (aTAAD), 10–20% are inoperable, with 50% not being amenable to ascending TEVAR due to the proximity of the intimal tear to the aortic root [72]. The Endo-Bentall is a device composed of a transcatheter aortic valve replacement (TAVR) and a TEVAR graft, with left and right coronary fenestrations/branches in the composite device. Numerous configurations have been described over time.

**Figure 11:**
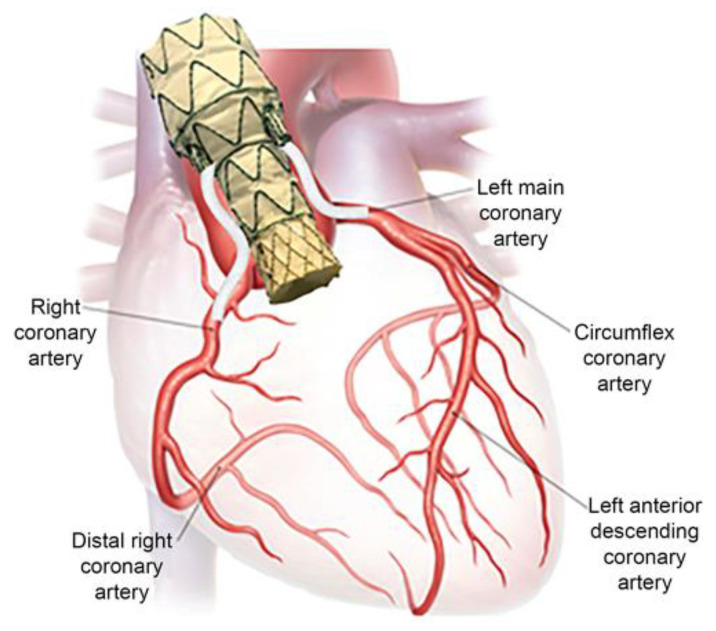
A depiction of the first in-human Endo-Bentall procedure [76].

The earliest report of an Endo-Bentall was by Gaia et al., who performed an Endo-Bentall in 2020 to treat a 64-year-old woman that developed a pseudoaneurysm around a previous open aortic valve replacement site. Their device was custom-made with a balloon-expandable valve connected to a TEVAR, as well as coronary branches (Figure 11). The main body of the device was delivered transapically under extracorporeal membrane oxygenation (ECMO) support and deployed, with the valve expanded via balloon angioplasty afterwards. The coronary stents were deployed via femoral access. The patient was alive at 9 months without symptoms and no endoleaks [76].

Gandet et al. treated an 82-year-old woman with a 10 cm symptomatic aneurysm of the distal arch and descending aorta, as well as a 5.5 cm ascending aortic aneurysm. They utilized three devices: (1) a modified TEVAR with three coronary artery fenestrations, (2) a balloon-expandable TAVR, and (3) a custom arch endograft with an innominate artery and left common carotid artery branch. Through-and-through wire access was obtained from transapical and transfemoral access. The modified ascending aorta TEVAR was deployed first, then the transcatheter aortic valve, and lastly the custom arch endograft. The procedure was performed under ECMO support and rapid pacing. Post-operatively, the patient was weaned off ECMO but developed a left-sided stroke requiring rehabilitation on discharge. On follow-up, there was a type Ia endoleak of the ascending aorta TEVAR but a complete seal of the arch endograft [77].

A third Endo-Bentall case was reported on by Leshnower et al. They presented a 71-year-old male with a fistula from the noncoronary sinus of Valsalva to the left atrium secondary to healed endocarditis. In their case, a modified TEVAR graft with coronary fenestrations was created and deployed first across the aortic valve. VBX stents were positioned into the coronary arteries but not deployed. The TAVR was then advanced and deployed, and finally, so were the coronary stents. Lastly, a Conformable TAG Thoracic Endoprosthesis was deployed to distally seal the original stent graft. This was all performed under cardiopulmonary bypass. Their case was complicated by the detachment of the right coronary stent, requiring ECMO and a new bridging stent. The patient was discharged on post-operative day 23, and on follow-up imaging, the graft and stents were all patent [78].

In 2024, Ghoreishi et al. presented five patients treated with the Endo-Bentall technique, four of whom with aTAAD and one with aortic root aneurysms. In this technique, a TEVAR and self-expanding TAVR were sutured together with coronary fenestrations made via cautery and then resheathed into a TEVAR delivery system. The device was delivered via femoral access under rapid pacing without cardiopulmonary bypass. The technical success rate was 100%. Two devices were used with coronary fenestrations, while three were used with coronary stents. Two of the five patients required a total endo-arch repair as well. There was one case of a permanent pacemaker insertion due to complete heart block. On the 3-month follow-up, there were no endoleaks or aortic insufficiency. There was one mortality although this was not aortic-related [79].

These papers represent an exciting new development in endovascular aortic surgery and demonstrate the possibility of an endovascular treatment option for all parts of the aorta.

## 9. Conclusions

The endovascular and hybrid repair of the aortic arch has seen a rise in novel technologies and the literature over the last decade. Interdisciplinary innovators in interventional radiology, cardiology, cardiothoracic surgery, and vascular surgery have globally pushed the boundaries of what can be accomplished through endovascular techniques. While long-term outcomes are pending and issues like technological limitations and high stroke/endoleak rates still need to be resolved, there have been promising early results. Ultimately, endovascular techniques seek to benefit the sickest patients who are not amenable to open repair, and their role in the aorta will only continue to grow.

## Data Availability

No new data were created or analyzed in this study.
